# Supplementary material for: Bismuth Doping in Nanostructured Tetrahedrite: Scalable Synthesis and Thermoelectric Performance
Source: Nanomaterials (Basel). 2021 May 25;11(6):1386. doi: 10.3390/nano11061386 (PMC8225167; doi:10.3390/nano11061386)
Supplement: Supplementary file 1 [file nanomaterials-11-01386-s001.zip › nanomaterials-1201627-supplementary.pdf]

# Supplementary Materials

# Bismuth doping in nanostructured tetrahedrite: Scalable synthesis and thermoelectric performance

**Peter Baláž [1], Emmanuel Guilmeau [2], Marcela Achimovičová [1,*], Matej Baláž [1], Nina, Daneu [3], Oleksandr Dobrozhan [4] and Mária Kaňuchová [5]**

1 Institute of Geotechnics, Slovak Academy of Sciences, 04353 Košice, Slovakia; balaz@saske.sk (P.B); achimovic@saske.sk (M.A.); balazm@saske.sk (M.B.)
2 CRISMAT, CNRS, Normandy University, ENSICAEN, UNICAEN, 14000 Caen, France; emmanuel.guilmeau@ensicaen.fr
3 Jozef Stefan Institute, SI-1000 Ljubljana, Slovenia; nina.daneu@ijs.si
4 Sumy State University, 40007 Sumy, Ukraine; dobrozhan.a@gmail.com
5 Institute of Earth Resources, Technical University Košice, 04001 Košice, Slovakia; maria.kanuchova@tuke.sk
* Correspondence: achimovic@saske.sk; Tel.: +421 557922607

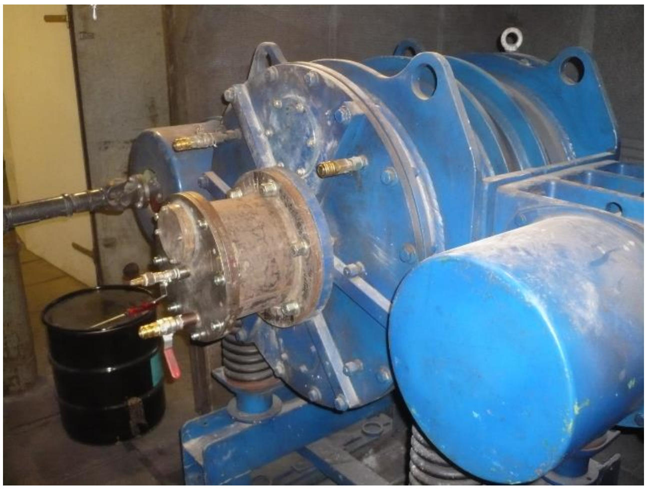

**Figure S1.** Eccentric industrial vibratory mill with attached satellite milling chamber.

## 1. Unreacted Sb and Bi inclusions

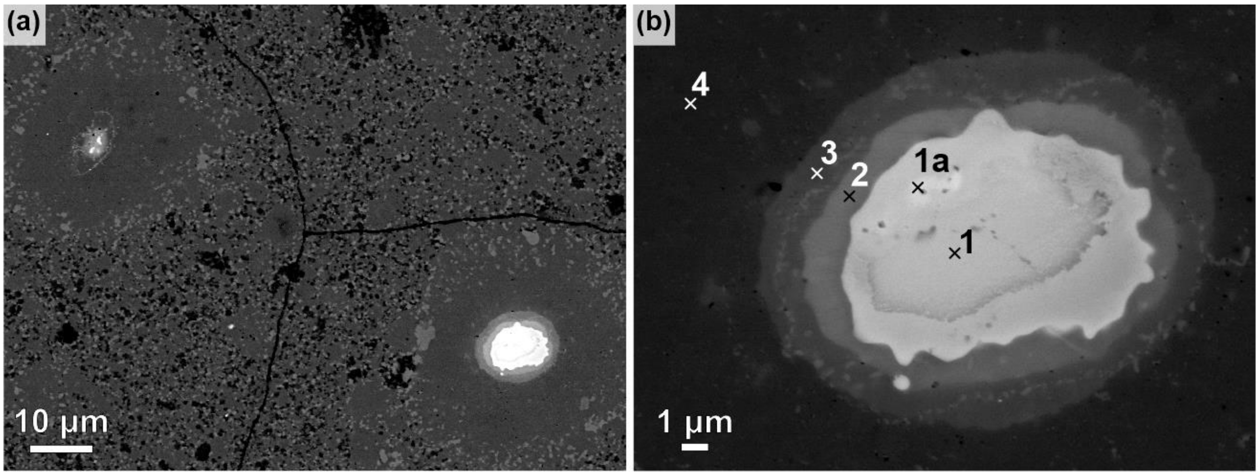

**Figure S2.** (a) Sample area with two partially reacted Sb-inclusions. (b) SEM/EDS analysis of the right particle has revealed that the brightest part is mostly metallic Sb with some Bi and perhaps also Cu. The XPS signal of elemental Bi probably stems from these unreacted particles, which probably formed by alloying of Sb with Bi and Cu during high-energy milling prior to the SPS densification. The texture radiating from the central part of the particle (marked by dashed line) formed during SPS processing. 1-4 mark the EDS point analyses, the results are shown in Table S1.

Table S1. Results of SEM/EDS analyses of the Sb/Bi inclusion area showing compositional zoning around the particle that formed during SPS.

| Spectrum | S, at% | Cu, at% | Sb, at% | Bi, at% |
|---|---|---|---|---|
| 1 | 0 | 3.2 | 86.2 | 10.5 |
| 1a | 0 | 4.2 | 78.3 | 17.5 |
| 2 | 54.8 | 6.0 | 34.1 | 5.0 |
| 3 | 49.1 | 24.1 | 24.7 | 2.1 |
| 4 | 46.1 | 37.8 | 15.1 | 0.9 |

## 2. Selected area electron diffraction of tetrahedrite

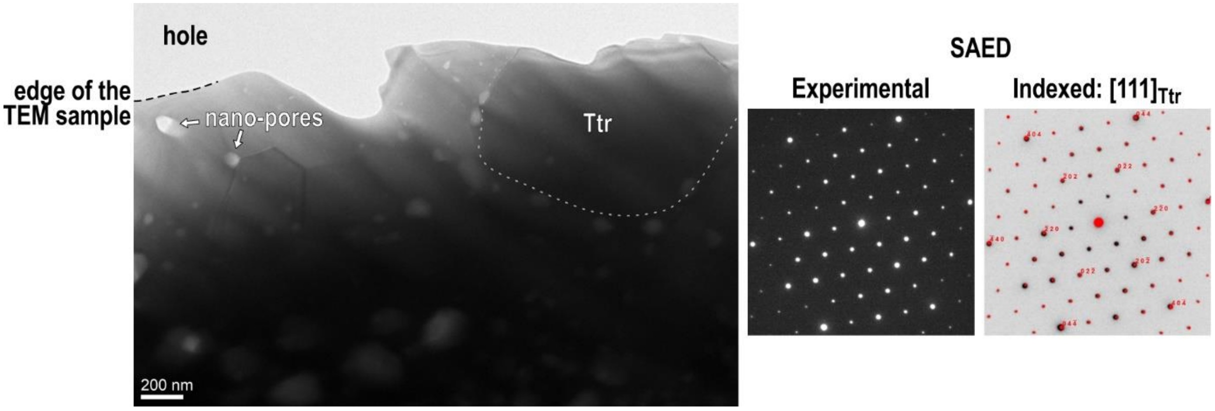


(a) (b)

**Figure S3.** (a) Conventional TEM image with marked edge of the TEM sample, hole (brightest part of the image) and nano-pores in the thin part of the sample. Tetrahedrite (Ttr) grain oriented along the [111] zone axis is marked in the image as revealed from (b) experimental and indexed SAED patterns.
